# Five-year follow up of genotypic resistance patterns in HIV-1 subtype C infected patients in Botswana after failure of thymidine analogue-based regimens

**DOI:** 10.1186/1758-2652-12-25

**Published:** 2009-10-25

**Authors:** Florence Doualla-Bell, Tendani Gaolathe, Ava Avalos, Suzanne Cloutier, Ndwapi Ndwapi, Christina Holcroft, Howard Moffat, Diana Dickinson, Max Essex, Mark A Wainberg, Madisa Mine

**Affiliations:** 1 McGill University AIDS Centre, Jewish General Hospital, Montreal, Quebec, Canada; 2 Botswana-Harvard School of Public Health, AIDS Initiative Partnership for HIV Research and Education, Gaborone, Botswana; 3 Infectious Disease Care Clinic, Princess Marina Hospital, Gaborone, Botswana; 4 Ministry of Health of Botswana, Botswana; 5 Statistical Consultation Service, Centre for Clinical Epidemiology and Community Studies, Jewish General Hospital Montreal, Quebec, Canada; 6 Independence Surgery, Gaborone, Botswana; 7 Harvard School of Public Health, Boston, Massachusetts, USA

## Abstract

**Objective:**

Our objective was to establish genotypic resistance profiles among the 4% of Batswana patients who experienced virologic failure while being followed within Botswana's National Antiretroviral Treatment Program between 2002 and 2007.

**Methods:**

At the beginning of the national program in 2002, almost all patients received stavudine (d4T), together with didanosine (ddI), as part of their first nucleoside reverse transcriptase inhibitor (NRTI)-based regimen (Group 1). In contrast, the standard of care for all patients subsequently enrolled (2002-2007) included zidovudine/lamivudine (ZDV/3TC) (Group 2). Genotypes were analyzed in 26 patients from Group 1 and 37 patients from Group 2. Associations between mutations were determined using Pearson's correlation coefficient and Jaccard's coefficient of similarity.

**Results:**

Seventy-eight percent of genotyped patients possessed mutations associated with protease inhibitor (PI) resistance while 87% and 90%, respectively, exhibited mutations associated with NRTIs and non-nucleoside reverse transcriptase inhibitors (NNRTIs). The most frequent PI mutations involving resistance to NFV were L90M (25.2%) and D30N (16.2%), but mutations at positions K45Q and D30N were often observed in tandem (P = 60.5, J = 50; *p *= 0.002; Group 2) alongside Q61E in 42.8% of patients who received ZDV/3TC. Both major patterns of thymidine analogue mutations, TAM 1 (48%) and TAM 2 (59%), were represented in patients from Group 1 and 2, although M184V was higher among individuals who had initially received ddI (61% versus 40.5%). In contrast, L74V was more frequent among individuals from Group 2 (16.2% versus 7.7%). Differences in regard to NNRTI mutations were also observed between Group 1 and Group 2 patients.

**Conclusion:**

Despite a low rate of therapeutic failure (4%) among these patients, those who failed possessed high numbers of resistance mutations as well as novel resistance mutations and/or polymorphisms at sites within reverse transcriptase and protease.

## Background

Botswana has an approximate 30% seroprevalence for HIV-1 [1] and was also one of the first African countries to provide universal free access to antiretroviral therapy (ART) in 2002. Prior to 2002, most patients in Botswana initiated therapy with d4T/ddI-based regimens. From January 2002, the main first-line regimen in Botswana was ZDV/3TC plus either efavirenz (EFV) or nevirapine (NVP) in women in whom pregnancy was likely to occur. For patients failing initial regimens, the main second-line regimen was d4T/ddI plus nelfinavir (NFV). More recently, lopinavir/ritonavir (LPV/r) has been approved for use in second-line to replace NFV, while tenofovir (TDF)/3TC is now used as a nucleoside reverse transcriptase inhibitor (NRTI) backbone in first-line [2].

By March 2007, 84,927 patients had entered the national ART programme, while an additional 9,514 had been followed in the private sector. The percentage of patients receiving therapy is estimated to be greater than 80% of the total need [3]. As of April 2007, a total of 17,400 patients had been registered at the Adult Infectious Disease Care Clinic (IDCC) located at the Princess Marina Hospital in Gaborone. Among these, approximately 11,000 were receiving comprehensive HIV treatment and care. This site has also launched a treatment failure protocol that has ascertained the treatment failure rate among ARV patients to be 4% [4].

Both acquired [5] and transmitted [6] drug resistance represent obstacles for the long-term use of ARVs. Previous publications from the IDCC described the frequencies and patterns of mutations in the reverse transcriptase (RT) and protease (PR) genes [7-9]. In general, no major impact of HIV subtypes has been observed regarding drug susceptibility [10,11].

We previously demonstrated that d4T/ddI-based regimens were associated with high rates of selection of the K65R mutation in HIV-1 subtype C [8,12]. For purposes of this analysis, patients failing therapy have been divided into those who received d4T/ddI as part of their first regimen (Group 1) versus those who initiated therapy with ZDV/3TC and who received ddI as part of second or subsequent regimens (Group 2). We also attempted to delineate associations between different mutations that are involved in drug resistance, as has already been calculated in subtype B viruses [13-15].

## Methods

### Patients and HIV-1 subtype C sequencing

Among the 4% of Batswana patients who experienced treatment failure at the IDCC, 363 received a ddI-based regimen as part of their first (Group 1) or second or subsequent regimen (Group 2). As recommended by the Botswana national programme since March 2005, all patients experiencing virological failure after their second-line regimen, mostly containing NFV as a first PI were evaluated by genotyping, *viz.*, a total of 63 patients (27 in Group 1 and 36 in Group 2).

These genotypes were linked to patients' demographic, immunologic, virologic and therapeutic clinical data. HIV genotyping for drug resistance was performed on plasma samples using a commercially available kit (ViroSeq HIV-1 genotyping system; Abbott Laboratories). All the patients were confirmed as possessing subtype C viruses on the basis of nucleotide sequence alignment using the ClustalX-version 1.8 sequencing tool [16]. The neighbour-joining method was employed for phylogenetic tree building using the NJplot programme.

### Statistical and mutational covariate analysis

We first calculated the frequencies of RT and PR mutations in the *pol *sequences of 26 Group 1 and 37 Group 2 patients who were followed. We also established relationships between the presence of specific mutations using both the Pearson correlation coefficient (P) and the Jaccard similarity coefficient (J). A Fisher's exact test was also performed to assess the significance of any associations that were found. As a reference, we employed the 2008 IAS-USA list of mutations [17].

## Results

### Patient characteristics

The characteristics of the 63 patients followed in this study are shown in Table 1, with virologic failure being defined as the inability to suppress plasma viremia or the rebound of viral load (VL) on at least two consecutive occasions to levels >400 copies RNA/ml. Median CD4 cell counts at failure were 334 cells/mm^3 ^for Group 1 and 233 cells/mm^3 ^for Group 2 and medians of viral loads (log10 HIV-1 RNA copies/ml) were 4.2 and 4.7 for Group 1 and 2, respectively, at which time most patients had received three different treatment regimens.

**Table 1:** Characteristics of the 63 patients who received ddI/d4T (Group 1) versus ZDV/3TC (Group 2) as first-line therapy

	Group 1 (n = 26)	Group 2 (n = 37)
*Age (years)* Median Interquartile range	35 [24-57]	34 [21-48]
		
*Female sex (%)*	61.5	60.7
		
*Baseline nadir CD4 cell count* Median Interquartile range	96 [2-276]	61 [1-317]
		
*CD4 cell count at failure visit* Median Interquartile range	334 [41-937]	233 [9-1313]
		
*Log10 HIV-1 RNA at failure visit* Median Interquartile range	4.2 [2.9-5.33]	4.7 [3.5-5.9]
		
*Total days on antiretroviral therapy at the time of genotype* Median Interquartile range	998 [228-2507]	486 [59-1346]
		
*Total time (days) to non-detectability of viral load (<400 copies/ml) at the time of genotype* Median Interquartile range	158 [28-1693]	96 [10-1188]
		
*Average number of ARV regimens received by the time of genotype*	3*	2.5^†^

*Group 1 - ARV regimens received by the time of genotyping. ddI/d4T/NVP, ZDV/3TC/EFV, ddI/d4T/NFV; ddI/d4T/NVP, ZDV/3TC/NVP, d4T/3TC/NVP, ABC/ddI/NFV; ddI, ddI/d4T/NVP; ddI/ZDV, ddI/d4T/EFV, ZDV/3TC/NFV; ddI/ZDV/3TC, ZDV/3TC/NVP, ABC/ddI/NFV; ddI/d4T/EFV, ZDV/3TC/EFV, ddI/d4T/NFV; ddI/d4T/NVP, ZDV/3TC/NFV (n = 2); ddI/d4T, ZDV/3TC/NVP, ddI/TDF/LPVr; ddI/d4T/EFV, ZDV/3TC/EFV (n = 3); ddI/ZDV, ZDV/3TC/NVP, ddI/d4T/NFV; ddI/d4T/NVP, ZDV/3TC/NFV, LPVr/SQV; ddI/ZDV/NVP, 3TC/d4T/NFV; ddI/d4T, ZDV/3TC/EFV, ddI/d4T/SQV/RTV; ddI/d4T/NVP, ZDV/3TC/NFV, ZDV/TDF/LPVr; ddI/ZDV/NVP, 3TC/d4T/IDV/RTV; ddI/d4T/EFV, ddI/d4T/NFV; ddI/d4T, ddI/d4T/NVP, ZDV/3TC/EFV; ddI/d4T/NVP, ZDV/3TC/NVP; ddI/d4T, 3TC/ddI, ZDV/3TC/NVP, ZDV/3TC/EFV; ddI/d4T, ZDV/3TC/SQV/RTV, ZDV/3TC/NVP; ddI/d4T/EFV, ZDV/3TC/EFV, ABC/ddI/NFV, ddI/d4T/EFV/LPV; ddI/d4T/EFV, ABC/ddI/NFV, d4T/3TC/EFV; ddI/ZDV/NVP, d4T/3TC/IDV, ddI/d4T/NFV.

^†^Group 2 - ARV regimens received by the time of genotyping. ZDV/3TC/NVP; ZDV/3TC/NVP, ddI/d4T/NVP, ZDV/3TC/NVP, 3TC/ddI/NFV, 3TC/ABC/ddI/LPV; ZDV/3TC/NVP, ddI/d4T/NFV (n = 13); ZDV/SQV/RTV, ddI/d4T, ddI/d4T/NFV, 3TC/ABC/LPV; ZDV/3TC/EFV, ddI/TDF/LPV; ZDV/3TC/IDV, ZDV/3TC/NVP, ddI/d4T/NVP; ABC/ddI/NVP; 3TC/d4T/NVP, ZDV/ddI/NFV; ZDV/3TC/NVP, ddI/d4T/NFV, ZDV/3TC/LPV; ZDV/3TC/EFV, ddI/d4T/NFV (n = 5); ZDV/3TC/NVP, ddI/d4T/NFV, ABC/SQV/RTV; ZDV/3TC/NVP, ddI/d4T/NFV, ABC/ddI/NFV; ZDV/3TC, SQV/RTV, ddI/d4T/SQV/RTV, ABC/d4T/ddI/LPV, 3TC/ddI/EFV; 3TC/d4T/EFV, ZDV/3TC/NFV; 3TC/d4T/EFV (n = 2); ZDV/3TC/EFV, 3TC/d4T/NVP; ZDV/3TC/EFV, ABC/ddI/NFV; ZDV/3TC/EFV (n = 2); ZDV/3TC, IDV/NVP, 3TC/ABC/d4T, d4T/EFV/IDV/RTV, 3TC/ABC/d4T, 3TC/ABC/d4T/LPV, 3TC/ABC/AZT/LPV.

### Mutations to protease inhibitors

**Figure 1:**
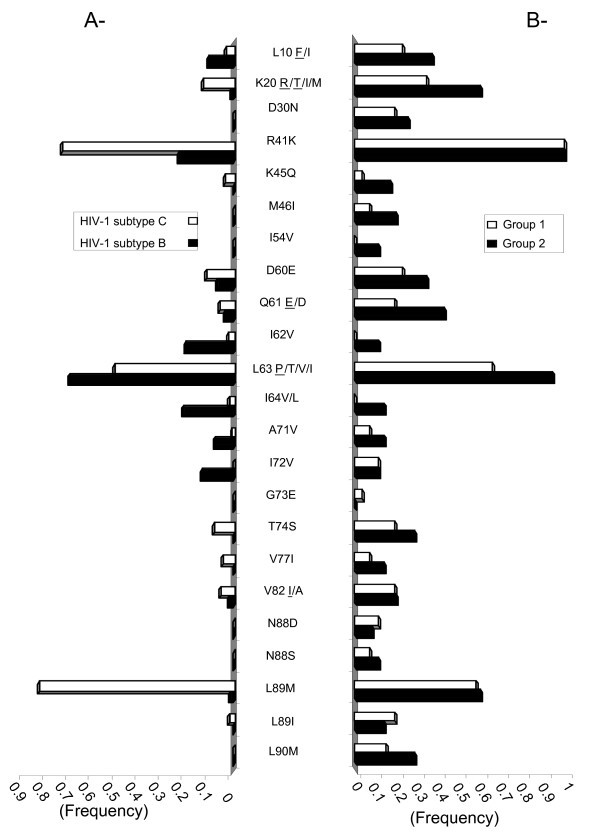
**Frequency of HIV-1 subtype C protease mutations**. **A **- The frequency of amino acid mutations in HIV-1 subtype C (white) or B (black) protease in drug-naïve patients are reported based on the Stanford HIV drug resistance database. **B **- Amino acids in HIV-1 subtype C protease of Batswana treated patients from Group 1 (white) or Group 2 (black) are reported. Where more than one amino acid change may have occurred, the most prevalent amino acid is underlined.

Phylogenetic analysis indicated that all of the 63 patients who were genotyped possessed subtype C viruses. Approximately 78% of PI-treated individuals exhibited resistance mutations; similar patterns of resistance were observed among both groups (Figure 1). Among patients who received NFV, the most common mutations observed were D30N and L90M, consistent with previous results (Table 2).

**Table 2:** Frequency of mutations within PR

Mutations	Frequency (%)		*P value
	**Group 1**	**Group 2**	

L10F/I	23	37	0.23

K20R/T/I/M	34.6	60	0.07

D30N	19.2	25.7	0.43

E35D/N	50	60	0.31

K45Q	3.8	17.14	0.12

M46I	7.7	20	0.19

D60E	23	34.28	0.43

Q61E/D	19	42.8	0.06

I62V	0	11.4	0.11

N88D	11.5	8.6	0.48

N88S	7.7	11.4	0.51

L89M	57.7	57.1	0.51

L89I	19.2	14.3	0.39

L90M	15	28	0.34

*P value: probability of significant differences between groups

Interestingly, 43% of patients possessed a Q61E mutation (CAA → GAA) that emerges from a silent substitution, i.e., CAA in subtype C relative to CAG in subtype B. The I62V resistance mutation was present in 11% of those patients who only received NFV as a PI. We confirmed the correlation seen by others between D30N and N88D (P = 74 and 52.4, J = 60 and 33.3; *p *= 0.004 and 0.01 for Groups 1 and 2, respectively) and also observed a novel association between D30N and K45Q (P = 60, J = 50; *p *= 0.002; Group 2), while L89I was only found in association with D30N (P = 50.5, J = 50; *p *= 0.002; Group 2) (Table 3).

**Table 3:** Significant correlations of pairs of mutations within PR

Mutations	Correlated mutations		Pearson coeff.		Jaccard coeff.		*P *value (freq cell)	
	**Group 1**	**Group 2**	**Group 1**	**Group 2**	**Group 1**	**Group 2**	**Group 1**	**Group 2**

L10F/I	K20R/T/I/M	M46I	36.9	36.7	36.4	33.3	0.13 (15.4)	0.07 (13.5)
	D30N	A71V	42.8	37.1	37.5	28.5	0.06 (11.5)	0.04 (10.8)
	Q61E/D		42.8		37.5		0.06 (11.5)	
	T74S	T74S	65.9	44.4	57.1	43.7	0.005 (15.4)	0.02 (0.02)
	N88D	N88D	65.9	40.36	50	23	0.008 (11.5)	0.04 (8.1)
	N88S		52		33		0.05 (7.7)	

K20R/T/I/M	E35D/N	E35D/N	40.4	50.1	46.7	65.4	0.10 (26.9)	0.006 (45.9)
	M46I	K45Q	39.7	38.4	0	28.5	0.11 (7.7)	0.03 (16.2)
	T74S		46.5				0.03 (15.4)	
	V77I		39.7				0.11 (7.7)	
	N88S		39.7				0.11 (7.7)	

D30N	K45Q	K45Q	41	60.5	20	50	0.19 (3.85)	0.002 (13.5)
	N88D	N88D	74	52.4	60	33.3	0.004 (11.5)	0.01 (8.1)
	L89I		50.5		42.8		0.03 (11.5)	

E35D/N	T74S		48.8		38.5		0.04 (19.2)	

M46I		A71V		41.5		33.3	0.04 (8.1)	
		V77I		41.5		33.3	0.04 (8.1)	

D60E		Q61E/D		48.6		50	0.005 (24.32)	

Q61E/D	N88S		59		40		0.03 (7.7)	

### Mutations to reverse transcriptase inhibitors

**Figure 2:**
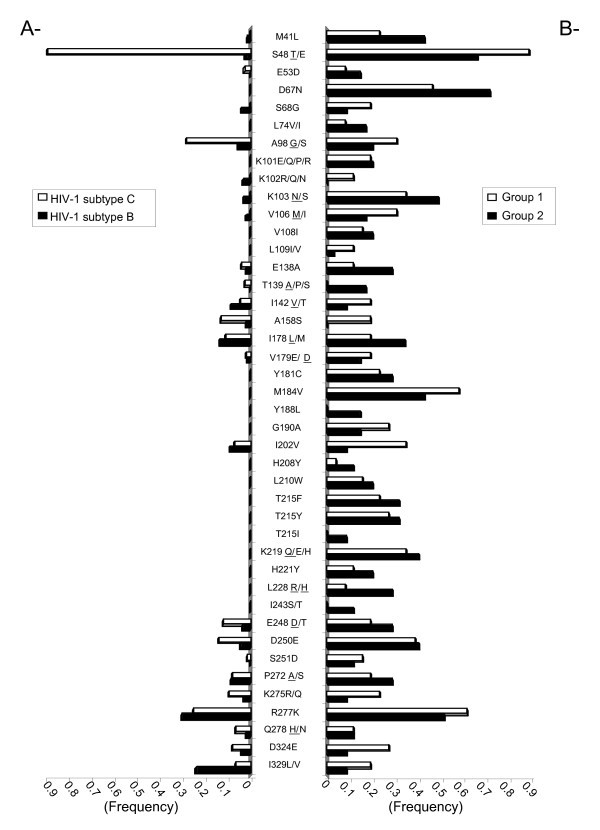
**Frequency of HIV-1 subtype C reverse transcriptase mutations**. **A **- The frequency of amino acid mutations in HIV-1 subtype C (white) or B (black) reverse transcriptase in drug-naïve patients are reported based on the Stanford HIV drug resistance database. **B **- Amino acids in HIV-1 subtype C reverse transcriptase of Batswana treated patients from Group 1 (white) or Group 2 (black) are reported. Where more than one amino acid change may have occurred, the most prevalent amino acid is underlined.

Approximately 87% of the patients who were genotyped possessed mutations associated with resistance to NNRTIs, while 90% presented with mutations associated with NRTIs (Table 4, Figure 2). Some individuals in Group 2 possessed I202V, L228R/H and D324E substitutions. M184V was observed in 16 of 26 Group 1 patients, 14 of whom had received 3TC prior to genotyping. In patients from Groups 1 and 2, both TAM 1 (48%) and TAM 2 (59%) mutations were present.

**Table 4:** Frequency of mutations within RT.

Mutations	Frequency (%)		*P value
	**Group 1**	**Group 2**	

**NNRTI**			

A98S/G	26.9	18.9	-

K101E/Q/P/R	19.23	21.6	-

K103N	34.6	48.6	-

V106M	30.8	16.21	-

V108I	15.4	18.9	-

E138A	15.4	27	-

V179E/D	15.3	13.5	-

Y181C	23	27	-

Y188L	0	13.5	-

G190A	23	13.5	-

**NRTI (non-TAM)**			

K65R	7.7	0	-

S68G	19.2	8.1	-

L74V/I	7.7	16.2	-

Q151M	3.8	2.7	-

M184V	61	40.5	-

**TAM1**			

41L	26.9	40.5	-

210W	19.2	18.9	-

215Y	26.9	29.7	-

**TAM2**			

67N	46	67.56	0.07

70R	35	37.8	-

215F	26.9	29.7	-

219Q	34.6	37.8	-

**Others**			

E53D	11.5	13.5	-

K102R/Q/N	15.4	0	-

I135V/T	30.8	45.9	-

T139A/P/S	0	16.2	-

I142V/T	15.4	8.1	-

A158S	23	0	-

S162C/A/T	15.4	24.3	-

I178L/M	19	35	-

I202V	34.6	8	0.008

E203A/K/D	3.8	18.9	-

H221Y	11.5	18.9	-

L228R/H	8	27	0.05

D250E	42.3	40.5	-

K275R/Q	23	8	0.09

D324E	27	8	0.04

*P value: probability of significant differences between groups

Similarly, 10 of 15 Group 2 patients who possessed M184V had been on a 3TC-containing regimen at the time of testing. Only two patients carried the K65R mutation; one of these had received TDF and the other had received ddI at the time of genotyping. The length of time on ineffective regimens (based on non-suppressed VL) was 809 days and 279 days for Groups 1 and 2, respectively.

Patients with the L74V mutation (16.2% in Group 2) were on ABC/ddI at the time of genotyping. In the group of patients who received EFV, the most frequent mutations were K103N, V106M and I135V/T, while Y181C and G190A were more frequent among patients who received NVP. In Group 2 patients, the Y181C and A98S/G mutations were found in association with the L228R/H mutation, [(J = 66.6, *p *= 0.0001) and (J = 41.7, *p *= 0.009), respectively], while L228R/H was associated with G190A in Group 1 patients (Table 5). We also observed an association between H221Y and Y181C in Group 1 patients treated with NVP.

**Table 5:** Significant correlations of pairs of mutations within RT.

Group 1	Pearson coeff.	Jaccard coeff.	*P *value	Group 2	Pearson coeff.	Jaccard coeff.	*P *value
101E/190A	65.92	57.1	0.005	101E/190A	36.8	30	0.06
101E/219Q	46.55	40	0.034	106M/139A	40.32	33.3	0.04
102R/53D	51.3	40	0.05	106M/138A	39.3	33.3	0.04
103N/178L	46	40	0.03	106M/179E	46.9	37.5	0.02
106M/283I	63.9	50	0.005	108I/208Y	49.85	37.5	0.02
190A/69D	65.9	50	0.008	181C/67N	42.2	40	0.01
				181C/69D	64.9	50	0.001
				181C/98S	63.8	54.5	0.001
142V/158S	52.5	42.8	0.03	181C/228R	72.6	66.6	0.0001
178L/135V	52.5	44.4	0.02				
221Y/162C	51.3	40	0.05	190A/41L	47.9	33.3	0.007
221Y/181C	66	50	0.008	190A/210W	41.5	33.3	0.04
228R/190A	52.7	33.3	0.05	190A/215Y	43.5	33.3	0.02
251D/53D	58.4	60	0.004	190A/74L	46.9	37.5	0.02
278N/60I	62.3	50	0.03				
283I/102R	70.4	60	0.006	228R/98S	48	41.7	0.009
294T/102R	67.7	50	0.02	228R/215F	40	40	0.04
				210W/215Y	44.07	38.4	*ND*
							
210W/215Y	36.4	33.3	*ND*	41L/215Y	42.64	44.4	*ND*
41L/215Y	41.3	40	*ND*	41L/210W	44.4	37.5	*ND*
41L/210W	36.4	33.3	*ND*	41L/53D	31.7	25	*ND*
41L/53D	59.5	42.8	*ND*	41L/74V/I	53	40	*ND*
67N/70R	62.37	61.5	*ND*	67N/70R	54.05	56	*ND*
70R/219Q	83.01	80	*ND*	70R/219Q	65.53	64.7	*ND*
219Q/67D	62.37	61.5	*ND*	219Q/67D	54.05	56	*ND*
215F/67D	40.85	35.7	*ND*	215F/67D	45.06	44	*ND*
215F/70R	36.9	33.3	*ND*	215F/70R	34.6	38.9	*ND*
219Q/69N/D	49.64	33.3	*ND*	219Q/69N/D	34.36	27	*ND*

## Discussion

This study provides data on drug resistance mutational profiles in HIV-1 subtype C-infected Batswana patients who experienced treatment failure between 2002 and 2007. Among novel findings, we have shown that resistance to NFV in these patients seems to involve the K45Q mutation in association with D30N, and occurs with similar frequency (J = 50, *p *= 0.002) as that previously characterized between D30N and N88D (J = 33.3, *p *= 0.01). The L89I mutation was also associated with D30N, suggesting that it may occur early in the establishment of resistance to NFV. Accumulation of large numbers of PI mutations may reflect the duration of time that patients may have been left on a failing regimen [18].

We previously demonstrated a greater propensity for patients infected by HIV-1 subtype C to develop the K65R mutation after treatment with ddI-based regimens in first-line therapy [8]. More recently, high rates of K65R were also observed among patients from West Africa who received 3TC/d4T/NNRTI as a first-line regimen [19,20]. High rates of virologic failure have also been reported in patients with the K65R mutation compared to L74V [21,22]. Both the L74V and K65R mutations may impact on subsequent TDF responses in patients who had previously received ddI [22].

Clearly, these two nucleoside cross-resistance mutations may compromise a wide array of future nucleoside-based therapeutic options in treatment-experienced patients. In the present study, the frequency of the K65R mutation was low (two of 26 patients). One possible explanation may be the reversion of the K65R mutation after use of subsequent ARV regimens, due to considerations of low viral fitness. This phenomenon emphasizes the need for more sensitive assays that can be used for surveillance of resistance mutations.

The high frequency of NNRTI mutations is also disturbing and raises questions about their use in first-line regimens in developing countries, where financial constraints may often compel physicians to keep patients on failing regimens for longer than desirable. In this context, it might be interesting to conduct a study such as ACTG 5142 [23], which compared the use of EFV to a first-line PI, in settings where subtype C viruses are predominant.

We also observed several associations involving less common resistance mutations, such as L228R/H and Y181C (J = 66.6, *p *= 0.0001) and A98S/G (J = 41.7, *p *= 0.009) in Group 2 patients and L228R/H in association with G190A in Group 1. We further observed a preferential emergence of the H221Y mutation in Group1 NVP-treated patients, who first exhibited the Y181C mutation. Moreover, mutations at positions A98, K101, V106, E138, Y181 and G190 were discovered, and it is not yet known whether these substitutions will affect clinical responsiveness to new NNRTIs, such as etravirine (ETV), in HIV-1 subtype C infections [24]. A review article on the subject of HIV drug resistance mutations and viral subtypes has recently been published [25].

Switching from ZDV/3TC to d4T/ddI (Group 2) and subsequent maintenance on a suboptimal NVP-containing regimen may also have resulted in higher rates of association between G190A and TAMs or G190A and L74V. Others have shown the co-presence of L74V and Y181C in patients who failed a NVP-containing regimen [26].

Although the Botswana ARV national programme has tried to optimize ARV therapy and has changed first- and second-line regimens to TDF/FTC/NNRTI and ZT/3TC/LPV/r, respectively, our results remain relevant for other developing country settings that still use ineffective combinations in a context of poor adherence. Indeed, it is important to understand genotypic resistance patterns in developing countries where treatment options may be more limited than those that are available wealthier countries and in which different viral subtypes may predominate. Associations such as those between the D30N and K45Q mutations in PR have not previously been reported. It is likely that the use of suboptimal therapy may facilitate the emergence of novel resistance pathways and that this will become a major concern in resource-limited settings.

## Conclusion

HIV subtype C-infected patients in Botswana who began therapy with either d4T/ddI- or ZDV/3TC-based regimens and who failed therapy have developed drug resistance-associated mutations at high frequency. These mutations are associated with resistance against each of NRTIs, NNRTIs, and PIs and include several novel substitutions relative to those typically observed with subtype B viruses. Hopefully, the current use of improved treatment regimens in Botswana will mitigate both rates of treatment failure as well as the future development and transmission of HIV.

## Competing interests

The authors declare that they have no competing interests.

## Authors' contributions

FDB wrote the initial draft of this manuscript and was largely responsible for compilation and interpretation of results. TG and AA helped draft the paper and manage data. SC and CH contributed data to the paper, while NN, HM, and ME corrected the paper and provided supervision. MM directed the laboratory in which sequencing results were obtained. MAW corrected the manuscript, provided interpretation of data, and collated results.
